# Supplementary material for: Identification of non-canonical peptides with moPepGen
Source: Nat Biotechnol. 2025 Jun 16;44(4):568–73. doi: 10.1038/s41587-025-02701-0 (PMC12680078; doi:10.1038/s41587-025-02701-0)
Supplement: Supplementary file 2 — Reporting Summary [file 41587_2025_2701_MOESM2_ESM.pdf]

nature portfolio

Corresponding author(s): Chenghao Zhu, Thomas Kislinger, Paul C. Boutros

Last updated by author(s): May 7, 2025

Reporting Summary

Nature Portfolio wishes to improve the reproducibility of the work that we publish. This form provides structure for consistency and transparency in reporting. For further information on Nature Portfolio policies, see our Editorial Policies and the Editorial Policy Checklist.

Statistics

For all statistical analyses, confirm that the following items are present in the figure legend, table legend, main text, or Methods section.

n/a	Confirmed	
☐	☒	The exact sample size (*n*) for each experimental group/condition, given as a discrete number and unit of measurement
☐	☒	A statement on whether measurements were taken from distinct samples or whether the same sample was measured repeatedly
☐	☒	The statistical test(s) used AND whether they are one- or two-sided<br>*Only common tests should be described solely by name; describe more complex techniques in the Methods section.*
☒	☐	A description of all covariates tested
☐	☒	A description of any assumptions or corrections, such as tests of normality and adjustment for multiple comparisons
☐	☒	A full description of the statistical parameters including central tendency (e.g. means) or other basic estimates (e.g. regression coefficient) AND variation (e.g. standard deviation) or associated estimates of uncertainty (e.g. confidence intervals)
☐	☒	For null hypothesis testing, the test statistic (e.g. *F*, *t*, *r*) with confidence intervals, effect sizes, degrees of freedom and *P* value noted<br>*Give P values as exact values whenever suitable.*
☒	☐	For Bayesian analysis, information on the choice of priors and Markov chain Monte Carlo settings
☒	☐	For hierarchical and complex designs, identification of the appropriate level for tests and full reporting of outcomes
☒	☐	Estimates of effect sizes (e.g. Cohen's *d*, Pearson's *r*), indicating how they were calculated

Our web collection on statistics for biologists contains articles on many of the points above.

Software and code

Policy information about availability of computer code

Data collection	No software was used to download the data used in this publication.
Data analysis	• moPepGen is available on GitHub: https://github.com/uclahs-cds/package-moPepGen and PyPI: https://pypi.org/project/mopepgen (licensed under GPL-2.0)<br>• The non-canonical peptide calling pipeline is available on GitHub: https://github.com/uclahs-cds/pipeline-call-NonCanonicalPeptide<br>• The DNA processing metapipeline is available on GitHub: https://github.com/uclahs-cds/metapipeline-DNA and includes the following software: BWA-MEM2 (v2.2.1), Picard Tools (v2.27.4), GATK (v4.2.5.0 and v4.2.4.1), SAMtools (v1.15.1), MuTect2 (from GATK v4.5.0.0), VCFtools (v0.1.16), BCFtools (v1.9-1) and VEP (v104)<br>• RNA-seq data were processed using: STAR (v2.7.10b), SAMtools (v1.15.1), STAR-Fusion (v1.9.1), rMATS (v4.1.1), REDItools2 (v1.0.0) and CIRCexplorer2 (v2.3.8)<br>• Proteomics data were processed using: ProteoWizard MSConvert (3.0.21258), Comet (v2019.01r5), MSFragger (v3.3), X!Tandem (v2015.12.15), OpenMS (v3.0.0-1f903c0), DIA-NN (v1.8.1) and Novor (app.novor.cloud)<br>• Neoantigens were predicted using OptiType (v1.3.5) and MHCflurry (v2.0.6)<br>• PSM validation were done using pyOpenMS (v3.1.0) and Oktoberfest (v0.6.2)<br>• Data analysis was performed using: R (v4.0.3), BoutrosLab.plotting.general (v6.0.2), data.table (v1.14.0), Python (v3.8.10)

For manuscripts utilizing custom algorithms or software that are central to the research but not yet described in published literature, software must be made available to editors and reviewers. We strongly encourage code deposition in a community repository (e.g. GitHub). See the Nature Portfolio guidelines for submitting code & software for further information.

Data

Policy information about availability of data

All manuscripts must include a data availability statement. This statement should provide the following information, where applicable:

- Accession codes, unique identifiers, or web links for publicly available datasets
- A description of any restrictions on data availability
- For clinical datasets or third party data, please ensure that the statement adheres to our policy

The processed CCLE data are available at the DepMap portal (http://www.depmap.org). The raw WGS and WXS cell lines sequencing data are available at Sequence Read Archive (SRA) and European Genome-Phenome Archive (EGA) under access number PRJNA52338041 and EGAD0000100103990. The raw mass spectrometry proteomic data are publicly available without restrictions at the ProteomeXchange via the PRIDE partner repository under accession number PXD03030442 for cell lines, PXD03098337 for mouse strain C57BL/6N, and PXD01015491 for alternative protease and fragmentation analyses. The proteomic data for the five prostate tumour samples are freely available at UCSD's MassIVE database under accession number MSV00008155236, whereas their raw WGS and RNA-seq data are available at EGA under accession EGAS0000100090035. Proteomic data for the eight kidney tumour samples are freely available at Proteomic Data Commons (PDC) under accession number PDC00041144, whereas the genomic and transcriptomic data are available at Genomic Data Commons (GDC, Project: CPTAC-3, Primary Site: Kidney) with dbGaP accession number phs001287, generated by the National Cancer Institute's Clinical Proteomic Tumor Analysis Consortium (CPTAC).

Research involving human participants, their data, or biological material

Policy information about studies with human participants or human data. See also policy information about sex, gender (identity/presentation), and sexual orientation and race, ethnicity and racism.

Reporting on sex and gender	This study did not involve human participants.
Reporting on race, ethnicity, or other socially relevant groupings	This study did not involve human participants.
Population characteristics	This study did not involve human participants.
Recruitment	This study did not involve human participants.
Ethics oversight	This study did not involve human participants.

Note that full information on the approval of the study protocol must also be provided in the manuscript.

Field-specific reporting

Please select the one below that is the best fit for your research. If you are not sure, read the appropriate sections before making your selection.

☒ Life sciences ☐ Behavioural & social sciences ☐ Ecological, evolutionary & environmental sciences

For a reference copy of the document with all sections, see nature.com/documents/nr-reporting-summary-flat.pdf

Life sciences study design

All studies must disclose on these points even when the disclosure is negative.

Sample size	The genomic and proteomic data for 375 cell lines were obtained from the Cancer Cell Line Encyclopedia project (Nusinow et al., 2020, Cell). Proteomic data for the three mouse tissue samples were obtained from Giansanti et al., 2022, Nat Methods. Proteomic data of a tonsil tissue sample, analyzed using 10 enzyme-fragmentation methods were accquired from Wang et al., 2019, Mol Syst Biol. The genomic and proteomic data for the 5 primary prostate tumours were obtained from Sinha et al., 2019, Cancer Cell. This study did not explicitly derive experimental groups, therefor the sample sizes were not determined based on statistical calculation.
Data exclusions	Data were included based on their accessibility. The 375 cancer cell lines were selected based on the availability of both proteomic and genomic data. The three mouse tissues (liver, uterus, and cerebellum) were chosen to cover the greatest tissue variability. Prostate tumors were selected because they have genomic and transcriptomic data available, as well as proteomic data from two injection replicates.
Replication	This study did not generate new experimental data from patients, samples, or cell lines. All analyses were performed using existing datasets and based on biological replicates, defined as independent patients, tumors, or cell lines, as appropriate to each analysis.
Randomization	As noted previously, this study did not explicitly derive experimental groups, and as such, no randomization was implemented during data analysis.
Blinding	Blinding was not employed during data analysis because this study involved secondary analysis of publicly available, pre-existing datasets. The goal of the analyses was to demonstrate the capability of moPepGen in detecting non-canonical peptides, and no conclusions were drawn from comparisons between predefined groups of samples.

Reporting for specific materials, systems and methods

We require information from authors about some types of materials, experimental systems and methods used in many studies. Here, indicate whether each material, system or method listed is relevant to your study. If you are not sure if a list item applies to your research, read the appropriate section before selecting a response.

Materials & experimental systems

n/a	Involved in the study
☒	☐ Antibodies
☒	☐ Eukaryotic cell lines
☒	☐ Palaeontology and archaeology
☒	☐ Animals and other organisms
☒	☐ Clinical data
☒	☐ Dual use research of concern
☒	☐ Plants

Methods

n/a	Involved in the study
☒	☐ ChIP-seq
☒	☐ Flow cytometry
☒	☐ MRI-based neuroimaging

Plants

Seed stocks	N/A
Novel plant genotypes	N/A
Authentication	N/A
